# Multicomponent mineral–phytogenic supplementation reduces Ascaridia galli shedding and systemic mycotoxin biomarkers while improving health and productive performance in laying hens

**DOI:** 10.14202/vetworld.2026.4008-4024

**Published:** 2026-09-14

**Authors:** Nurgul Montayeva, Sarsenbek Montayev, Aizere Sagyzgaliyeva, Abzal Kereyev

**Affiliations:** 1 West Kazakhstan Agrarian and Technical University named after Zhangir Khan, Uralsk 090009, Republic of Kazakhstan.

**Keywords:** Artemisia absinthium, Ascaridia galli, antioxidant status, laying hens, mineral–phytogenic supplement, mycotoxin biomarkers, phytogenic feed additive, poultry health

## Abstract

**Background and Aim:**

Gastrointestinal helminthiasis and dietary mycotoxin exposure can concurrently impair poultry health and productivity, whereas conventional control strategies generally target these challenges separately. This study evaluated whether a multicomponent mineral–phytogenic supplement could simultaneously reduce *Ascaridia galli* shedding and systemic mycotoxin exposure while improving hematological, hepatic, antioxidant, and productive responses in naturally challenged laying hens.

**Materials and Methods:**

Fifty 26-week-old Lohmann Brown laying hens naturally infected with *A. galli* and exposed to feed naturally contaminated with aflatoxin B1 (AFB1), deoxynivalenol (DON), and T-2 toxin were randomly allocated to control and experimental groups (n = 25/group) for 30 days. Controls received the basal diet, whereas the experimental group received the basal diet supplemented at 170 g/kg with dried *Cucurbita maxima* pulp and peel, pumpkin seed meal, opoka, coquina meal, and post-frost *Artemisia absinthium*. Fecal and blood samples were collected on days 0, 10, 20, and 30. *A. galli* fecal egg counts, serum AFB1, deoxynivalenol-3-sulfate (DON-3S), T-2 toxin, hematological and hepatic indices, antioxidant biomarkers, and egg production and quality were evaluated.

**Results:**

Baseline measurements were comparable between groups. By day 30, expected *A. galli* shedding was 64.0% lower in supplemented hens than in controls (incidence rate ratio = 0.36; 95% confidence interval = 0.22–0.51; p < 0.001). Serum AFB1, DON-3S, and T-2 toxin concentrations were lower by 37.9%, 38.8%, and 41.9%, respectively. Supplemented hens showed increased erythrocyte counts, hemoglobin, hematocrit, total protein, albumin, superoxide dismutase, catalase, and glutathione peroxidase, together with decreased leukocyte counts, eosinophils, hepatic transaminases, and malondialdehyde. Hen-day production increased from 86.2% to 93.8%, daily egg mass from 49.9 to 57.0 g/hen, shell thickness from 0.350 to 0.395 mm, and yolk color from 6.4 to 10.5 Roche units. No mortality or adverse clinical signs occurred.

**Conclusion:**

The mineral–phytogenic supplement concurrently reduced *A. galli* shedding and systemic mycotoxin biomarkers while improving hematological, hepatic, antioxidant, and productive outcomes. These findings support further evaluation of this regionally sourced formulation as an integrated nutritional strategy for laying hens exposed to concurrent parasitic and mycotoxin challenges.

## INTRODUCTION

Gastrointestinal parasitism and dietary mycotoxicosis are major challenges to commercial poultry production worldwide [1, 2]. These stressors may occur concurrently under field conditions; in a survey of 122 broiler farms, every feed sample contained at least one mycotoxin, and 93% contained more than three [3]. Both gastrointestinal parasitism and mycotoxin exposure can compromise poultry health, welfare, and productive performance [1, 4].

*Ascaridia galli* is a macroscopically visible enteric nematode of considerable economic importance in poultry production. Its reported bird-level prevalence reaches 41.1%, with the highest prevalence observed in extensively reared birds with outdoor access [2]. Adult worms inhabit the small intestine and are associated with focal inflammation, mucosal hemorrhage, and ulceration, which have been observed in approximately half of infected birds examined at necropsy [2]. Parasite burdens are typically overdispersed within affected populations [5], and natural infection has been associated with reduced erythrocyte counts and eosinophilia [6]. The histotropic larval phase is also presumed to cause mechanical disruption of the intestinal villi; however, this effect has not been demonstrated directly in laying hens.

Aflatoxin B1 (AFB1) and deoxynivalenol (DON) are important feed-borne mycotoxins associated with hepatotoxicity, oxidative stress, and immunosuppression in poultry [1, 4, 7]. DON exerts substantial effects on the gastrointestinal tract; the European Food Safety Authority reported decreased jejunal villus height at dietary concentrations as low as 1.9 mg/kg and established a reference point of 0.6 mg/kg for poultry other than laying hens [8]. Concurrent intestinal injury caused by *A. galli* infection and DON exposure could potentially compromise intestinal barrier integrity and thereby influence systemic mycotoxin exposure. However, intestinal permeability was not measured in the present study; therefore, this potential interaction remains a mechanistic hypothesis rather than an experimentally demonstrated pathway.

Several natural materials have been investigated as potential alternatives or adjuncts to conventional parasite and mycotoxin control strategies. Seeds of *Cucurbita* spp. contain cucurbitine, a non-proteinogenic pyrrolidine amino acid, and pumpkin seed extracts have reduced fecal egg counts (FEC) and worm burdens in nematode-infected rodents, although the active principle responsible for these effects has not been conclusively established [9]. Piperazine, a chemically distinct anthelmintic, acts as a low-potency agonist at extrasynaptic gamma-aminobutyric acid receptors in ascarid muscle [10]. A comparable mechanism has been proposed for cucurbitine, but this mechanism has not been experimentally demonstrated. *Artemisia absinthium* contains several sesquiterpene lactones, including absinthin, anabsinthin, anabsin, and artabsin [11], whereas extracts from *Artemisia* spp. have been reported to alter antioxidant enzyme gene expression in nematode larvae [12]. Opoka, a carbonate-siliceous sedimentary rock, has also been evaluated as a dietary component in mycotoxin-exposed broilers [13] and in combination with a plant decoction in calves [14].

Current control practices generally address gastrointestinal helminthiasis and mycotoxin exposure separately. Periodic anthelmintic administration remains an important approach to parasite control, whereas feed-borne mycotoxins are commonly managed with adsorbents or binders [15]. However, access to effective parasite control interventions may be uneven [2], and concerns regarding anthelmintic resistance and drug residues in food-producing animals have increased interest in complementary nutritional strategies. Conventional single-agent mycotoxin binders also have physicochemical limitations because adsorption efficiency depends on properties such as charge distribution, polarity, pore size, and surface area. Consequently, an adsorbent surface with high affinity for relatively polar aflatoxins may not bind less polar trichothecenes such as DON as effectively. Furthermore, non-selective adsorption by some clay-based sorbents may decrease the availability of vitamins, amino acids, and minerals [16, 17].

Multicomponent mineral–phytogenic formulations may provide a broader strategy by combining materials with complementary physicochemical and biological properties. Previous reviews have described the potential of individual phytogenic compounds as anti-mycotoxin agents [16] and formulation technologies intended to improve phytogenic efficacy in pigs and poultry [18, 19]. Other studies have investigated nano-chitosan during combined AFB1 and fumonisin B1 exposure [20], combinations of additives that produced greater effects than individual agents on immune and gut microbial endpoints [21], and herbal formulations associated with increased antioxidant enzyme activities [22, 23]. Collectively, these findings provide a rationale for evaluating formulations designed to act simultaneously on different components of concurrent parasitic and toxicological challenges.

Despite these developments, an important research gap remains regarding the simultaneous management of gastrointestinal helminthiasis and dietary multi-mycotoxin exposure in laying hens. To our knowledge, no previous controlled study has evaluated a sedimentary mineral matrix combined with *Cucurbita maxima* and post-frost *A. absinthium* under concurrent natural *A. galli* infection and dietary exposure to multiple mycotoxins. Previous studies have largely investigated these challenges independently, including phytogenic anthelmintics in infected hens without documented concurrent mycotoxin exposure [22, 24] and mineral sorbents in mycotoxin-exposed broilers without a documented helminth burden [13]. Experimental models have also commonly relied on single-toxin supplementation or defined artificial parasite egg doses [24], which may not reproduce the naturally overdispersed distribution of helminth burdens [5] or the multiple-mycotoxin exposure profiles encountered in commercial poultry feeds [3].

A further methodological gap concerns the assessment of internal DON exposure in poultry. Parent DON undergoes rapid sulfation in avian species [25]; therefore, measuring the parent compound alone may provide an incomplete estimate of systemic exposure. Evaluation of deoxynivalenol-3-sulfate (DON-3S), together with circulating AFB1 and T-2 toxin, may therefore provide a more informative assessment of the systemic mycotoxin burden. An experimental model incorporating natural *A. galli* infection, naturally contaminated feed, longitudinal parasitological and systemic mycotoxin measurements, and complementary health and production outcomes could consequently provide a more integrated assessment of a multicomponent nutritional intervention.

This study hypothesized that combining a porous sedimentary mineral fraction with complementary phytogenic components would produce concurrent responses to gastrointestinal helminth infection and dietary mycotoxin exposure in laying hens. The primary objective was to determine the effects of a multicomponent mineral–phytogenic supplement on *A. galli* fecal egg shedding; systemic concentrations of AFB1, T-2 toxin, and DON-3S; and hematological, hepatic, and antioxidant responses in naturally challenged laying hens. Productive performance and egg quality responses were also evaluated to determine whether changes in parasitological, toxicological, and physiological endpoints were accompanied by measurable production-related effects.

The secondary objectives were to characterize the mineral fraction physicochemically and to evaluate a formulation based on regionally available raw materials, comprising Taskala opoka, Mangystau coquina meal, locally grown *C. maxima* cv. ‘Mramornaya’, and post-frost *A. absinthium*. Characterization of the mineral components and standardization of the geographically defined plant materials were intended to improve formulation reproducibility and provide a defined basis for subsequent evaluation of this multicomponent nutritional strategy.

## MATERIALS AND METHODS

### Ethical approval

All experimental procedures involving laying hens were reviewed and approved by the Academic Council of Zhangir Khan West Kazakhstan Agrarian and Technical University (Approval No. 7/21/02/2025). The study was conducted in accordance with applicable national directives governing the humane care, handling, housing, and use of animals in agricultural research. The experimental design, animal allocation, husbandry, outcome assessment, and reporting were conducted in accordance with the ARRIVE 2.0 guidelines [26]. Throughout the 30-day experimental period, birds were monitored for clinical abnormalities, feed refusal, and other adverse events.

### Study period and location

The 30-day experiment in laying hens was conducted from June 2 to July 2, 2025, at the Veterinary Clinic of the Institute of Veterinary and Agrotechnology, Zhangir Khan West Kazakhstan Agrarian and Technical University, Uralsk, Republic of Kazakhstan.

### Study design

This study was a controlled, 30-day, parallel-group feeding trial involving 50 Lohmann Brown laying hens naturally infected with *A. galli* and exposed to dietary mycotoxins. The hens were randomly allocated to an unsupplemented control group (n = 25) or a supplemented experimental group (n = 25). The control group received the basal diet alone, whereas the experimental group received the same basal diet supplemented with the mineral–phytogenic formulation at 170 g/kg basal diet. Blood and fecal samples were collected from individual birds on days 0, 10, 20, and 30.

Hens were eligible for inclusion if they were clinically healthy on veterinary examination, were 26 weeks of age and at the same production stage, originated from the same flock, and were shedding *A. galli* eggs at screening. No post-allocation exclusion criteria were applied. No birds were lost to follow-up, and no data points were excluded from the analyses [26].

### Animal housing, randomization, and blinding

Fifty 26-week-old Lohmann Brown laying hens with natural gastrointestinal helminth infection were randomly allocated to the unsupplemented control group (n = 25) or supplemented experimental group (n = 25). Simple randomization was performed using a computer-generated random-number sequence applied to individual wing-band numbers. The group size of 25 hens was determined by the number of age-matched birds available from a single flock and the available cage capacity.

Birds were housed individually in wire cages in a climate-controlled facility maintained at 20–22°C under a 16-h light/8-h dark photoperiod. Individual housing permitted measurement of feed intake and fecal output for each bird. Because each cage contained one hen, the individual bird and cage represented the same experimental unit, and all measured endpoints were attributable to the same 50 individual hens.

Eggs were collected and counted daily. Hen-day egg production was calculated for each bird over the 7 days preceding each sampling point. Egg weight, shell thickness, shell breaking strength, and yolk color were determined using all eggs produced by each hen over three consecutive days on days 0 and 30, with up to three eggs assessed per hen and the resulting measurements averaged for each bird. Shell thickness was measured at the equator after removal of the shell membrane using a digital micrometer (Mitutoyo Corp., Kawasaki, Japan). Shell breaking strength was determined by quasi-static compression using an Egg Force Reader (ORKA Food Technology Ltd., Ramat Hasharon, Israel), and yolk color was assessed using the Roche yolk color fan (F. Hoffmann-La Roche Ltd., Basel, Switzerland).

Feed batches were coded by an individual who was not otherwise involved in the study. The technician performing FEC, the liquid chromatography–tandem mass spectrometry (LC-MS/MS) operator, and personnel conducting hematological, biochemical, and antioxidant analyses remained blinded to treatment allocation until database lock.

### Dietary formulation and feed preparation

A corn–soybean meal basal diet was formulated to meet National Research Council recommendations for laying hens. Baseline LC-MS/MS analysis confirmed natural contamination of the diet with 20.4 µg/kg AFB1, 5.1 mg/kg DON, and 34.2 µg/kg T-2 toxin. When expressed at 12% moisture, the AFB1 concentration marginally exceeded the maximum permitted concentration of 0.02 mg/kg for complete feedingstuffs for poultry other than young animals specified in Directive 2002/32/EC [27]. The DON concentration marginally exceeded the guidance value of 5 mg/kg for complementary and complete feedingstuffs established in Recommendation 2006/576/EC [28]. The concentration of T-2 toxin alone was approximately one-seventh of the 0.25 mg/kg indicative level established for the combined concentration of T-2 and HT-2 toxins in compound feed under Recommendation 2013/165/EU [29].

The experimental supplement comprised dried pumpkin pulp and peel powder (10%, w/w, as-fed basis), dried pumpkin seed meal (3%), opoka from the Taskala deposit, West Kazakhstan Oblast (1.5%), coquina meal (1.5%), and dried *A. absinthium* (1.0%). The basal diet provided 2,748 kcal metabolizable energy (ME)/kg and 16.5% crude protein, whereas the experimental diet provided 2,652 kcal ME/kg and 16.2% crude protein (Table 1). Feed and water were provided *ad libitum*. The potential sorbent capacity of opoka was inferred from its measured surface area and porosity and from previously published *in vivo* findings in broilers [13]. No feed refusal or selective feeding was observed, and daily dry matter intake did not differ between groups.

### Raw material preparation

*Cucurbita maxima* Duchesne cv. ‘Mramornaya’ fruits were obtained from a single local producer within one harvest season. The fruits were washed, halved, and separated into pulp, peel, and seeds. The pulp and peel fraction was sliced to a thickness of 5–10 mm and dried at 40–50°C in a forced-convection oven (Binder ED, Binder GmbH, Tuttlingen, Germany) until the residual moisture content was ≤10%. Moisture content was verified gravimetrically according to GOST 31640-2012 [30] and using an infrared moisture analyzer (KERN DBS, KERN & Sohn GmbH, Balingen, Germany). Water activity was <0.60, as determined using an AquaLab 4TE (METER Group, Pullman, WA, USA). The dried pulp and peel fraction and seeds were milled separately using a Retsch ZM 200 mill (Retsch GmbH, Haan, Germany) through 1.0- and 0.5-mm sieves, respectively.

Opoka was jaw-crushed to 15–20 mm and dried at 100–150°C until residual moisture content was ≤7%. This temperature range was selected to remove free water while remaining below temperatures associated with the loss of surface silanol groups through condensation. The dried material was subsequently ball-milled to a particle size of <1.0 mm. X-ray diffraction identified an amorphous opal-CT phase with superimposed quartz reflections. The Brunauer–Emmett–Teller (BET) surface area was 64 m²/g, porosity was 43%–47%, and bulk density was 1,200–1,350 kg/m³. Characterizing the mineral by measurable physicochemical properties rather than a trade name facilitates comparison with commercial mineral binders authorized for use in poultry feed, including bentonite [31], and improves formulation reproducibility. Coquina meal (~95% CaCO₃) obtained from Mangystau Oblast was ground to a particle size of <1.0 mm.

Aerial parts of *A. absinthium* L. were collected after the first autumn frosts in West Kazakhstan Oblast. Harvest timing was used as a standardization criterion for the phytogenic fraction, analogous to the use of deposit origin and BET surface area for characterization of the mineral fraction. The concentrations of phytochemical constituents in *A. absinthium* vary according to phenological stage; thujone content decreases by >30% at full bloom compared with earlier stages, absinthin increases from the bud stage to early flowering and subsequently decreases at full bloom, and artemisetin decreases by approximately 25% from the bud stage to full bloom [11]. The plant material was dried at 40–50°C to a moisture content of ≤10% and sequentially processed using a hay chopper, grain crusher, and ball mill to obtain a particle size of <1.0 mm in accordance with GOST ISO 6498-2014 [32]. All prepared powders were stored under nitrogen at 4°C until use.

**Table 1:** Ingredient composition of the experimental diets.

**Ingredient**	**Basal diet (both groups), g/kg as-fed basis**
Corn, yellow grain	551
Soybean meal (44% CP)	252
Sunflower oil	33
Wheat bran	35
Limestone (CaCO₃, 38% Ca)	87
Monocalcium phosphate	15
NaCl	3.5
Vitamin–mineral premix	10
DL-Methionine (99%)	2.0
L-Lysine HCl (78%)	1.0
Choline chloride (60%)	0.5
Kaolin (carrier/filler)	10
**Total**	**1,000**
**Experimental supplement**	**Added to experimental group only,** **g/kg basal diet**
Dried pumpkin pulp and peel powder (≤10% moisture, 1.0 mm)	100
Dried pumpkin seed meal (≤10% moisture, 0.5 mm)	30
Opoka (SiO₂–CaCO₃, Taskala deposit, 64 m²/g, porosity 43%–47%)	15
Coquina meal (CaCO₃, Mangystau deposit)	15
Dried A. absinthium L. (1.0%)	10
**Total supplement**	**170**

Values are expressed as g/kg on an as-fed basis. CP = Crude protein.

### Parasitological quantification and negative binomial modeling

Individual fresh fecal samples were collected on days 0, 10, 20, and 30 and maintained at 4°C for no longer than 6 h before processing. FEC were determined using the modified McMaster flotation technique with saturated salt solution at a specific gravity of 1.20. The method had an analytical sensitivity of 50 eggs per gram of feces (EPG). Eggs were identified as *A. galli* by light microscopy based on their ovoid morphology and smooth, thick shell, consistent with the reported dimensions of 72–92 × 45–57 µm.

Each preparation was additionally examined for *Heterakis* *gallinarum*, *Capillaria* spp., *Trichostrongylus tenuis*, and cestode taxa. No eggs attributable to these taxa and no cestode proglottids were detected at any sampling point.

To account for the intrinsic overdispersion of helminth egg count data, individual EPG measurements were analyzed using a negative binomial generalized linear mixed model (NB-GLMM) [5]. Treatment group and time were included as fixed effects, individual bird identity was included as a random intercept to account for repeated measurements within birds, and fecal dry weight was incorporated as an offset.

### Systemic biomonitoring of circulating mycotoxins using isotope-dilution LC-MS/MS

Peripheral blood samples were collected longitudinally into serum separator tubes on days 0, 10, 20, and 30. Serum was separated by centrifugation at 2,000 × g for 15 min and stored at −80°C until analysis.

Because avian presystemic metabolism rapidly and extensively biotransforms parent DON through hepatic sulfotransferase activity, measurement of the parent compound alone may provide an incomplete assessment of internal exposure [25]. Serum was therefore analyzed for DON-3S, which has been identified as a major DON metabolite in broiler chickens, pullets, roosters, and turkeys and has previously been quantified in excreta rather than serum [25], together with circulating free AFB1 and T-2 toxin.

Analytes were quantified by isotope-dilution LC-MS/MS using a SCIEX QTRAP 6500+ (SCIEX, Framingham, MA, USA) coupled to a Shimadzu Nexera X2 (Shimadzu Corp., Kyoto, Japan) ultra-high-performance liquid chromatography system. Matrix-matched calibration with stable isotope-labeled internal standards (¹³C₁₇-AFB1, d₃-DON-3S, and d₃-T-2 toxin) was used to correct for matrix-dependent ionization. Analytes were extracted by protein precipitation optimized for low sample volumes. Free AFB1 additionally underwent C18 solid-phase extraction and immunoaffinity concentration to enable quantification in the low-pg/mL range.

The limits of quantification (LOQs) were 0.005 ng/mL for AFB1, 0.10 ng/mL for DON-3S, and 0.05 ng/mL for T-2 toxin. Limits of detection and LOQs corresponded to signal-to-noise ratios of 3:1 and 10:1, respectively, according to ICH Q2(R1). All reported serum AFB1 concentrations (12.3–19.8 pg/mL) exceeded the LOQ. Calibration was linear over concentration ranges of 0.005–1.00 ng/mL for AFB1, 0.10–20.0 ng/mL for DON-3S, and 0.05–10.0 ng/mL for T-2 toxin (r² > 0.996). Recovery against certified reference materials ranged from 91% to 106%. Intra-day and inter-day precision ranged from 4.2% to 6.5% and from 6.1% to 8.3%, respectively, and matrix effects were 92%, 104%, and 88% for the respective analytes.

### Hematological, biochemical, and antioxidant analyses

Hematological parameters, including erythrocyte count (RBC), leukocyte count (WBC), hemoglobin (Hb), hematocrit (Hct), and leukocyte differential counts, including the eosinophil fraction, were determined using an automated veterinary hematology analyzer (Mindray BC-2800Vet, Shenzhen Mindray Bio-Medical Electronics Co., Ltd., Shenzhen, China). Serum total protein, albumin, aspartate aminotransferase (AST), and alanine aminotransferase (ALT) were measured using a Mindray BS-380 biochemical analyzer (Shenzhen Mindray Bio-Medical Electronics Co., Ltd.). Albumin was quantified using the bromocresol green method.

Erythrocyte superoxide dismutase (SOD), catalase (CAT), and glutathione peroxidase (GPx) activities, expressed per milligram of protein, and serum malondialdehyde (MDA) concentrations were determined using commercial colorimetric kits (Nanjing Jiancheng Bioengineering Institute, Nanjing, China). The assays used kit A001-1-2 for SOD (hydroxylamine method), A007-1-1 for CAT (ammonium molybdate method), A005-1-2 for GPx (5,5′-dithiobis-(2-nitrobenzoic acid), and A003-1-2 for MDA (thiobarbituric acid-reactive substances method). MDA concentrations were expressed as nmol/mL. All assays were performed according to the manufacturer's instructions. Intra-assay and inter-assay coefficients of variation were ≤5.5% and ≤8.2%, respectively.

Total protein used for normalization was quantified by the Coomassie Brilliant Blue G-250 method using kit A045-2-1 (Nanjing Jiancheng Bioengineering Institute, Nanjing, China), with bovine serum albumin as the calibrator. Samples were analyzed in duplicate, and samples from all four time points for each bird were analyzed within the same run to minimize between-run analytical variation.

### Statistical analysis

Statistical analyses were performed using IBM SPSS Statistics version 27.0 (IBM Corp., Armonk, NY, USA) and R version 4.3.2 (R Core Team, Vienna, Austria). Baseline FEC values are presented as median and interquartile range (IQR), whereas continuous variables are presented as mean ± standard deviation (SD).

Repeated measurements were analyzed using mixed-effects linear models, with treatment, time, and the treatment × time interaction included as fixed effects and individual bird included as a random effect. Bonferroni-adjusted post hoc comparisons were performed where appropriate. FEC data were analyzed using an NB-GLMM implemented using the glmmTMB package, with treatment and time as fixed effects, individual bird as a random intercept, and fecal dry weight as an offset. Results are expressed as incidence rate ratios (IRRs) with 95% confidence intervals (CIs). The Benjamini–Hochberg procedure was applied to control the false discovery rate across predefined families of secondary endpoints. Statistical significance was set at p < 0.05.

## RESULTS

Across the six endpoint domains evaluated, supplementation showed a consistent pattern of responses during the 30-day experimental period. These included a 64.0% reduction in *A. galli* fecal egg shedding, decreases in all three circulating mycotoxin biomarkers, and improvements in erythroid, hepatic, antioxidant, and production-related parameters. All endpoints were assessed longitudinally in the same individually housed hens.

### FEC

Baseline fecal egg burdens were comparable between the groups (control: 1,280 EPG, IQR 850–1,810; experimental: 1,265 EPG, IQR 830–1,790; Table 2 and Figure 1). The NB-GLMM dispersion parameter was θ = 1.14. Under the nbinom2 parameterization implemented in glmmTMB, the variance is expressed as σ² = μ(1 + μ/θ) [33]. Accordingly, θ corresponds to the negative binomial dispersion exponent k commonly used in parasitological analyses [5]. The observed value indicated substantial overdispersion of the egg count data relative to a Poisson distribution and supported use of the negative binomial modeling framework.

Median fecal egg shedding in the control group remained relatively stable during the experimental period, increasing from 1,280 EPG on day 0 to 1,420 EPG on day 30. In contrast, the experimental group showed a progressive reduction in fecal egg shedding. Relative to the control group, expected shedding was 25% lower on day 10 (IRR = 0.75; 95% CI = 0.58–0.96), 48% lower on day 20 (IRR = 0.52; 95% CI = 0.36–0.74), and 64.0% lower on day 30 (510 vs. 1,420 EPG; IRR = 0.36; 95% CI = 0.22–0.51; p < 0.001). Thus, by day 30, the expected fecal egg count in supplemented hens was approximately 36% of that observed in controls.

**Table 2:** Ascaridia galli fecal egg counts over 30 days.

**Time point**	**Control (n = 25), median (IQR)**	**Experimental (n = 25), median (IQR)**	**IRR (95% CI)**
Day 0	1,280 (850–1,810)	1,265 (830–1,790)	1.00 (Ref.)
Day 10	1,340 (900–1,850)	1,010 (600–1,410)	0.75 (0.58–0.96)
Day 20	1,385 (920–1,890)	720 (450–980)	0.52 (0.36–0.74)
Day 30	1,420 (950–1,920)	510 (310–720)	0.36 (0.22–0.51)***

CI = Confidence interval; EPG = Eggs per gram of feces; IQR = Interquartile range; IRR = Incidence rate ratio; Ref. = Reference. ***p < 0.001.

**Figure 1:**
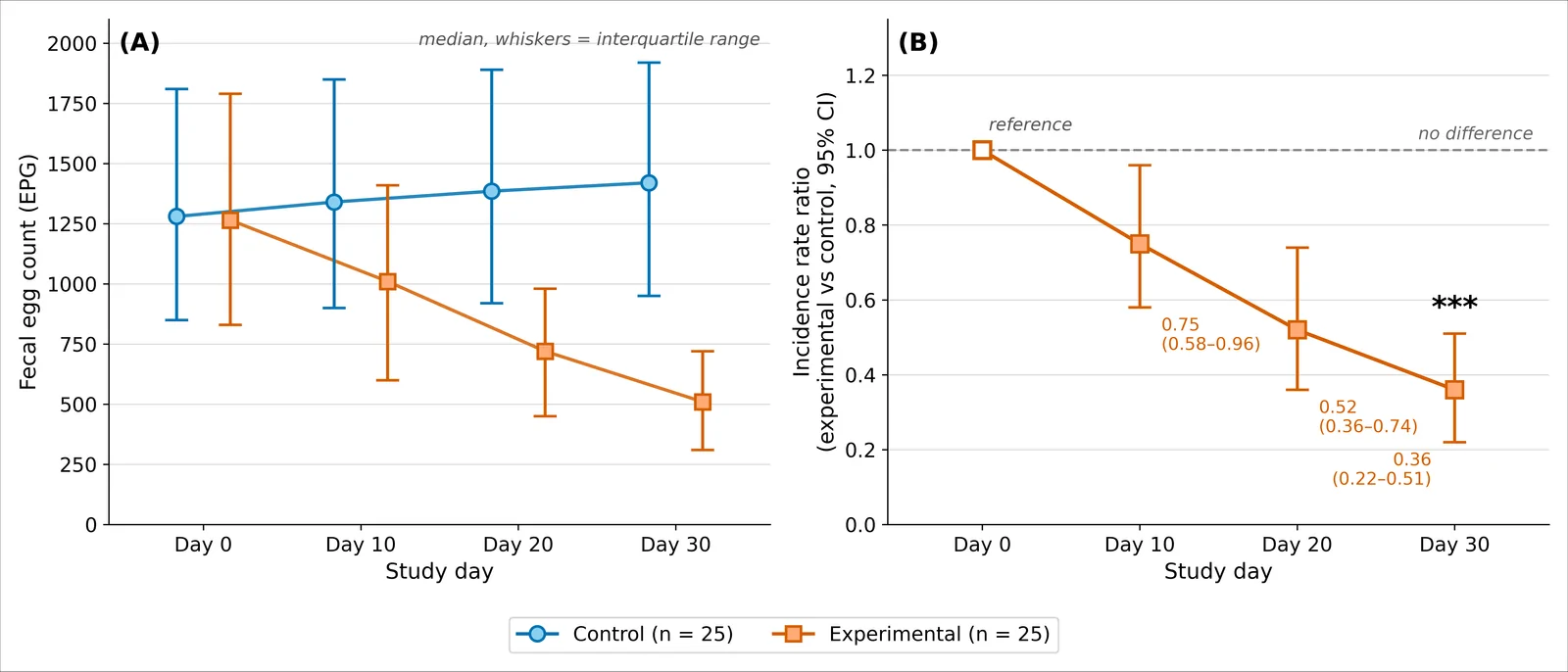
*Ascaridia galli* fecal egg count dynamics over 30 days.

### Mycotoxin biomarkers

LC-MS/MS analysis demonstrated progressive divergence in circulating mycotoxin biomarkers between the groups during the experimental period (Table 3 and Figure 2). Daily dry matter intake did not differ between the groups at any sampling point.

By day 30, the experimental group had a 37.9% lower serum AFB1 concentration than the control group (12.3 ± 3.2 vs. 19.8 ± 4.5 pg/mL; p = 0.003). DON-3S was 38.8% lower in the experimental group (4.10 ± 0.95 vs. 6.70 ± 1.30 ng/mL; p = 0.001), whereas T-2 toxin was 41.9% lower (50.0 ± 14.0 vs. 86.0 ± 20.0 pg/mL; p = 0.002). Concentrations in the control group remained relatively stable or increased slightly over time, whereas concentrations in the experimental group declined progressively from day 10 onward.

**Table 3:** Circulating mycotoxin biomarkers.

**Mycotoxin biomarker**	**Time point**	**Control (n = 25),** **mean ± SD**	**Experimental (n = 25),** **mean ± SD**	**p-value** **(group × time)**
AFB1 (pg/mL)	Day 0	18.4 ± 4.1	18.7 ± 3.9	—
	Day 10	19.1 ± 4.3	16.5 ± 3.6	—
	Day 20	19.5 ± 4.4	14.2 ± 3.4	—
	Day 30	19.8 ± 4.5	12.3 ± 3.2	0.003**
DON-3S (ng/mL)	Day 0	6.40 ± 1.20	6.45 ± 1.15	—
	Day 10	6.55 ± 1.25	5.60 ± 1.05	—
	Day 20	6.62 ± 1.28	4.80 ± 1.00	—
	Day 30	6.70 ± 1.30	4.10 ± 0.95	0.001***
T-2 toxin (pg/mL)	Day 0	82.0 ± 18.0	80.0 ± 17.0	—
	Day 10	84.0 ± 19.0	70.0 ± 15.0	—
	Day 20	85.0 ± 20.0	60.0 ± 14.0	—
	Day 30	86.0 ± 20.0	50.0 ± 14.0	0.002**

AFB1 = Aflatoxin B1; DON-3S = Deoxynivalenol-3-sulfate; SD = Standard deviation. **p < 0.01; ***p < 0.001.

**Figure 2:**
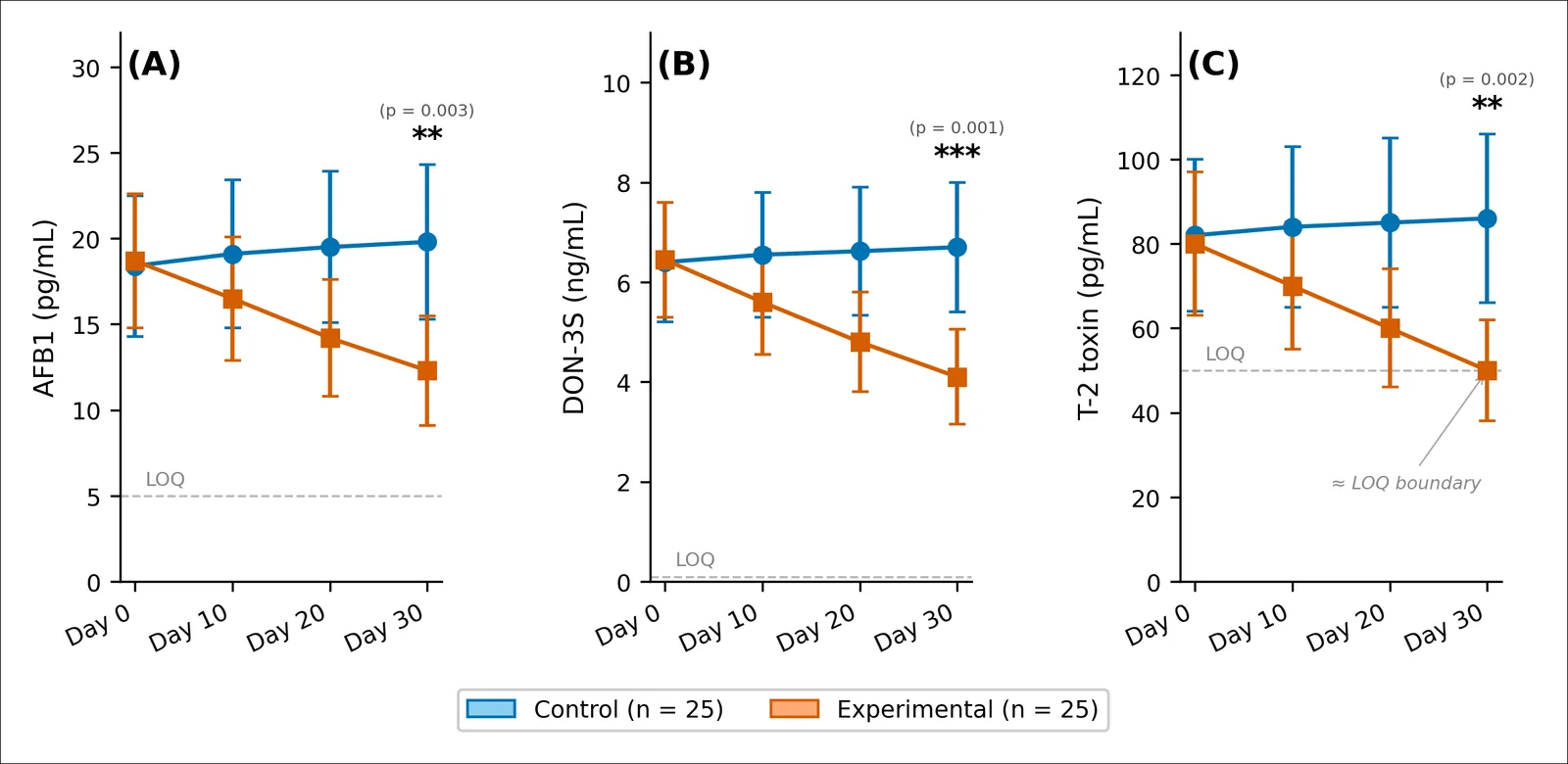
Circulating mycotoxin biomarkers.

### Hematological parameters

By day 30, the experimental group had higher RBC, Hb, and Hct values than the control group (Table 4 and Figure 3). RBC increased from 2.64 ± 0.30 × 10¹²/L on day 0 to 3.02 ± 0.31 × 10¹²/L on day 30 in the experimental group, whereas the corresponding control value was 2.64 ± 0.28 × 10¹²/L on day 30 (p = 0.012). Hb increased from 102 ± 11 to 117 ± 13 g/L in the experimental group compared with 102 ± 11 g/L in controls on day 30 (p = 0.018). Similarly, Hct increased from 31 ± 3.5% to 36 ± 3.8% in the experimental group compared with 31 ± 3.5% in controls on day 30 (p = 0.014).

WBC decreased from 18.8 ± 3.2 × 10⁹/L on day 0 to 16.1 ± 2.8 × 10⁹/L on day 30 in the experimental group, whereas the control group remained relatively stable at 18.8 ± 3.2 × 10⁹/L on day 30 (p = 0.022). The eosinophil proportion declined from 4.2 ± 1.1% to 2.3 ± 0.8% in the experimental group, compared with 4.2 ± 1.1% in controls on day 30 (p = 0.006). From baseline to day 30, the proportional reduction in eosinophils was 45.2%, compared with a 14.4% reduction in total WBC. The temporal decline in eosinophils occurred in parallel with the reduction in FEC across the four sampling points (Tables 2 and 4).

**Table 4:** Systemic hematological parameters over the experimental period.

**Parameter**	**Time point**	**Control (n = 25),** **mean ± SD**	**Experimental (n = 25),** **mean ± SD**	**p-value** **(group × time)**
RBC (×10¹²/L)	Day 0	2.63 ± 0.29	2.64 ± 0.30	—
	Day 10	2.62 ± 0.28	2.78 ± 0.29	—
	Day 20	2.61 ± 0.27	2.91 ± 0.30	—
	Day 30	2.64 ± 0.28	3.02 ± 0.31	0.012*
Hb (g/L)	Day 0	101 ± 10	102 ± 11	—
	Day 10	100 ± 11	108 ± 12	—
	Day 20	101 ± 10	112 ± 12	—
	Day 30	102 ± 11	117 ± 13	0.018*
Hct (%)	Day 0	30 ± 3.4	31 ± 3.5	—
	Day 10	30 ± 3.3	33 ± 3.6	—
	Day 20	31 ± 3.4	35 ± 3.7	—
	Day 30	31 ± 3.5	36 ± 3.8	0.014*
WBC (×10⁹/L)	Day 0	18.6 ± 3.1	18.8 ± 3.2	—
	Day 10	18.7 ± 3.2	17.5 ± 3.0	—
	Day 20	18.9 ± 3.3	16.8 ± 2.9	—
	Day 30	18.8 ± 3.2	16.1 ± 2.8	0.022*
Eosinophils (%)	Day 0	4.3 ± 1.2	4.2 ± 1.1	—
	Day 10	4.4 ± 1.1	3.6 ± 1.0	—
	Day 20	4.3 ± 1.1	2.9 ± 0.9	—
	Day 30	4.2 ± 1.1	2.3 ± 0.8	0.006**

Hb = Hemoglobin; Hct = Hematocrit; RBC = Red blood cell count; SD = Standard deviation; WBC = White blood cell count. *p < 0.05; **p < 0.01.

### Hepatic biochemical and antioxidant parameters

By day 30, AST and ALT activities were lower in the experimental group than in controls, whereas total protein and albumin concentrations were higher (Table 5 and Figure 4). AST was 160 ± 22 U/L in the experimental group compared with 180 ± 25 U/L in controls (p = 0.021), and ALT was 30 ± 5 versus 36 ± 6 U/L, respectively (p = 0.017). Total protein was 64 ± 7 g/L in the experimental group compared with 56 ± 6 g/L in controls (p = 0.009), whereas albumin was 28 ± 3 versus 24 ± 3 g/L, respectively (p = 0.015). Differences between the groups became progressively more evident from day 10 onward. Albumin values were interpreted as relative indices because albumin was quantified using the bromocresol green assay in avian serum.

Antioxidant enzyme activities also differed between groups by day 30 (Table 5 and Figure 5). Erythrocyte SOD activity was higher in the experimental group than in controls (133 ± 21 vs. 102 ± 18 U/mg protein; p = 0.004). Similarly, CAT activity was 73 ± 11 versus 58 ± 9 U/mg protein (p = 0.006), and GPx activity was 109 ± 15 versus 78 ± 12 U/mg protein (p = 0.001). In contrast, serum MDA concentration was lower in the experimental group than in controls on day 30 (4.2 ± 0.7 vs. 5.8 ± 0.9 nmol/mL; p = 0.002).

**Figure 3:**
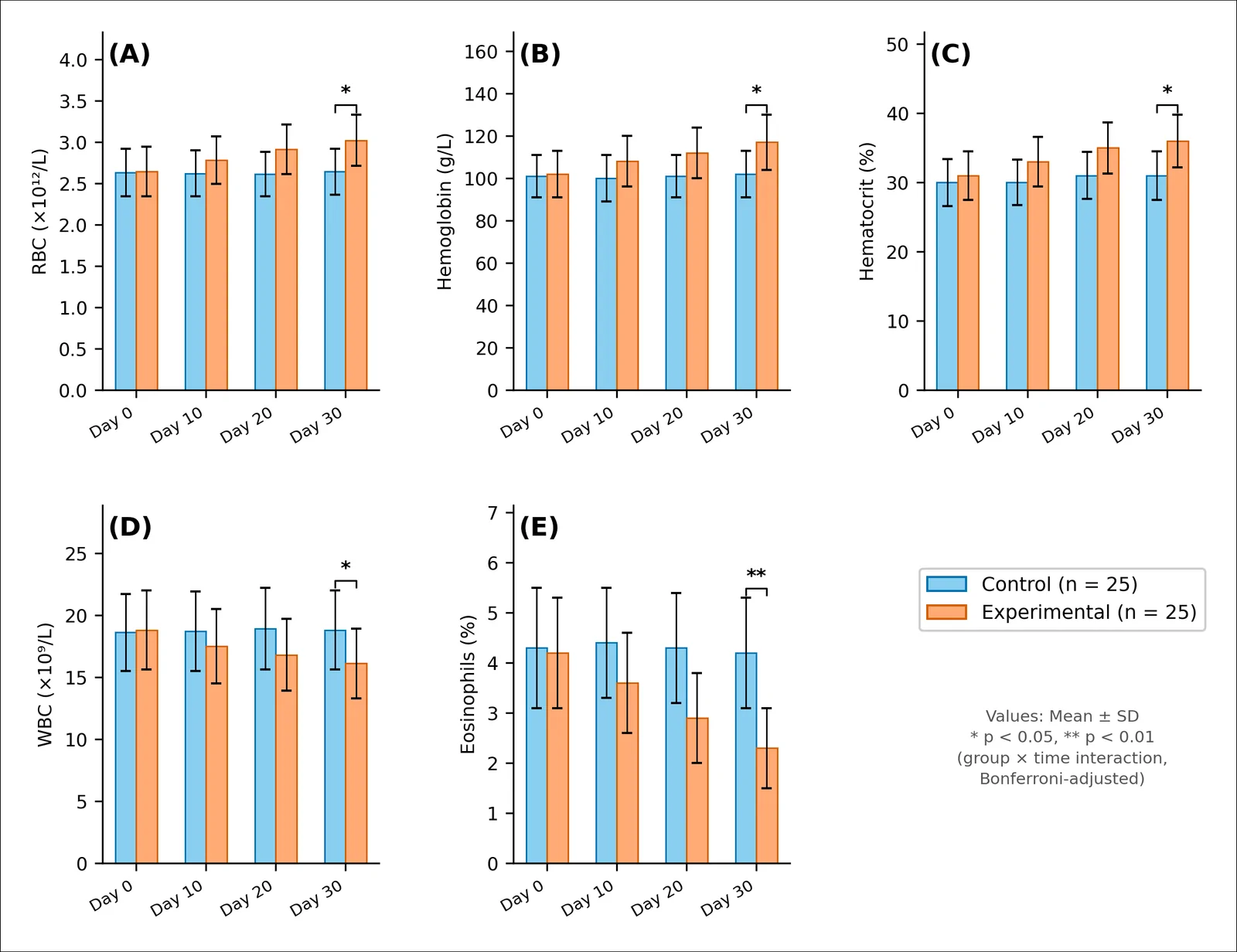
Systemic hematological parameters.

**Table 5:** Hepatic biochemical and antioxidant parameters.

**Parameter**	**Time point**	**Control (n = 25),** **mean ± SD**	**Experimental (n = 25),** **mean ± SD**	**p-value** **(group × time)**
AST (U/L)	Day 0	178 ± 24	180 ± 25	—
	Day 10	179 ± 25	172 ± 23	—
	Day 20	180 ± 25	165 ± 22	—
	Day 30	180 ± 25	160 ± 22	0.021*
ALT (U/L)	Day 0	35 ± 6	36 ± 6	—
	Day 10	36 ± 6	33 ± 5	—
	Day 20	36 ± 6	31 ± 5	—
	Day 30	36 ± 6	30 ± 5	0.017*
Total protein (g/L)	Day 0	55 ± 6	56 ± 6	—
	Day 10	55 ± 6	59 ± 6	—
	Day 20	56 ± 6	62 ± 7	—
	Day 30	56 ± 6	64 ± 7	0.009**
Albumin (g/L)†	Day 0	23 ± 3	24 ± 3	—
	Day 10	24 ± 3	26 ± 3	—
	Day 20	24 ± 3	27 ± 3	—
	Day 30	24 ± 3	28 ± 3	0.015*
SOD (U/mg protein)	Day 0	101 ± 17	102 ± 18	—
	Day 10	102 ± 18	115 ± 19	—
	Day 20	103 ± 18	125 ± 20	—
	Day 30	102 ± 18	133 ± 21	0.004**
CAT (U/mg protein)	Day 0	57 ± 8	58 ± 9	—
	Day 10	58 ± 9	65 ± 10	—
	Day 20	58 ± 9	70 ± 10	—
	Day 30	58 ± 9	73 ± 11	0.006**
GPx (U/mg protein)	Day 0	77 ± 11	78 ± 12	—
	Day 10	78 ± 12	92 ± 13	—
	Day 20	78 ± 12	101 ± 14	—
	Day 30	78 ± 12	109 ± 15	0.001***
MDA (nmol/mL)	Day 0	5.7 ± 0.9	5.8 ± 0.9	—
	Day 10	5.8 ± 0.9	5.1 ± 0.8	—
	Day 20	5.8 ± 0.9	4.6 ± 0.8	—
	Day 30	5.8 ± 0.9	4.2 ± 0.7	0.002**

ALT = Alanine aminotransferase; AST = Aspartate aminotransferase; CAT = Catalase; GPx = Glutathione peroxidase; MDA = Malondialdehyde; SD = Standard deviation; SOD = Superoxide dismutase. †Albumin was determined using the bromocresol green method and should be interpreted as a relative index in avian serum. *p < 0.05; **p < 0.01; ***p < 0.001.

### Egg production and egg quality

Egg production and egg quality parameters were comparable between groups on day 0 (Table 6). By day 30, hen-day production in the experimental group had increased from 86.2% to 93.8%, whereas the control group changed from 86.5% to 85.8% (p < 0.001 for the group × time comparison). Egg weight increased from 57.9 ± 1.3 to 60.8 ± 1.1 g in the experimental group compared with 58.2 ± 1.4 g in controls on day 30 (p < 0.001). Daily egg mass increased from 49.9 ± 1.9 to 57.0 ± 1.6 g/hen in the experimental group, whereas the corresponding control value was 49.9 ± 2.1 g/hen on day 30 (p < 0.001).

Shell thickness increased from 0.350 ± 0.014 mm on day 0 to 0.395 ± 0.010 mm on day 30 in the experimental group, compared with 0.348 ± 0.015 mm in controls on day 30 (p < 0.001). Shell breaking strength increased from 36.2 ± 2.0 to 44.5 ± 1.8 N in the experimental group, whereas the control value was 35.8 ± 2.4 N on day 30 (p < 0.001). Yolk color increased from 6.4 ± 0.4 to 10.5 ± 0.5 Roche units in the experimental group, compared with 6.2 ± 0.4 in controls on day 30 (p < 0.001).

**Table 6:** Egg production and egg quality parameters of Lohmann Brown laying hens over the 30-day experimental period.

**Parameter**	**Time point**	**Control (n = 25),** **mean ± SD**	**Experimental (n = 25),** **mean ± SD**	**p-value** **(group × time)**
Laying rate (%)	Day 0	86.5 ± 2.1	86.2 ± 2.3	—
	Day 30	85.8 ± 2.4	93.8 ± 1.5	<0.001***
Egg weight (g)	Day 0	57.8 ± 1.2	57.9 ± 1.3	—
	Day 30	58.2 ± 1.4	60.8 ± 1.1	<0.001***
Daily egg mass (g/hen)	Day 0	50.0 ± 1.8	49.9 ± 1.9	—
	Day 30	49.9 ± 2.1	57.0 ± 1.6	<0.001***
Shell thickness (mm)	Day 0	0.352 ± 0.012	0.350 ± 0.014	—
	Day 30	0.348 ± 0.015	0.395 ± 0.010	<0.001***
Shell breaking strength (N)	Day 0	36.5 ± 2.1	36.2 ± 2.0	—
	Day 30	35.8 ± 2.4	44.5 ± 1.8	<0.001***
Yolk color (Roche score)	Day 0	6.5 ± 0.5	6.4 ± 0.4	—
	Day 30	6.2 ± 0.4	10.5 ± 0.5	<0.001***

SD = Standard deviation. ***p < 0.001.

**Figure 4:**
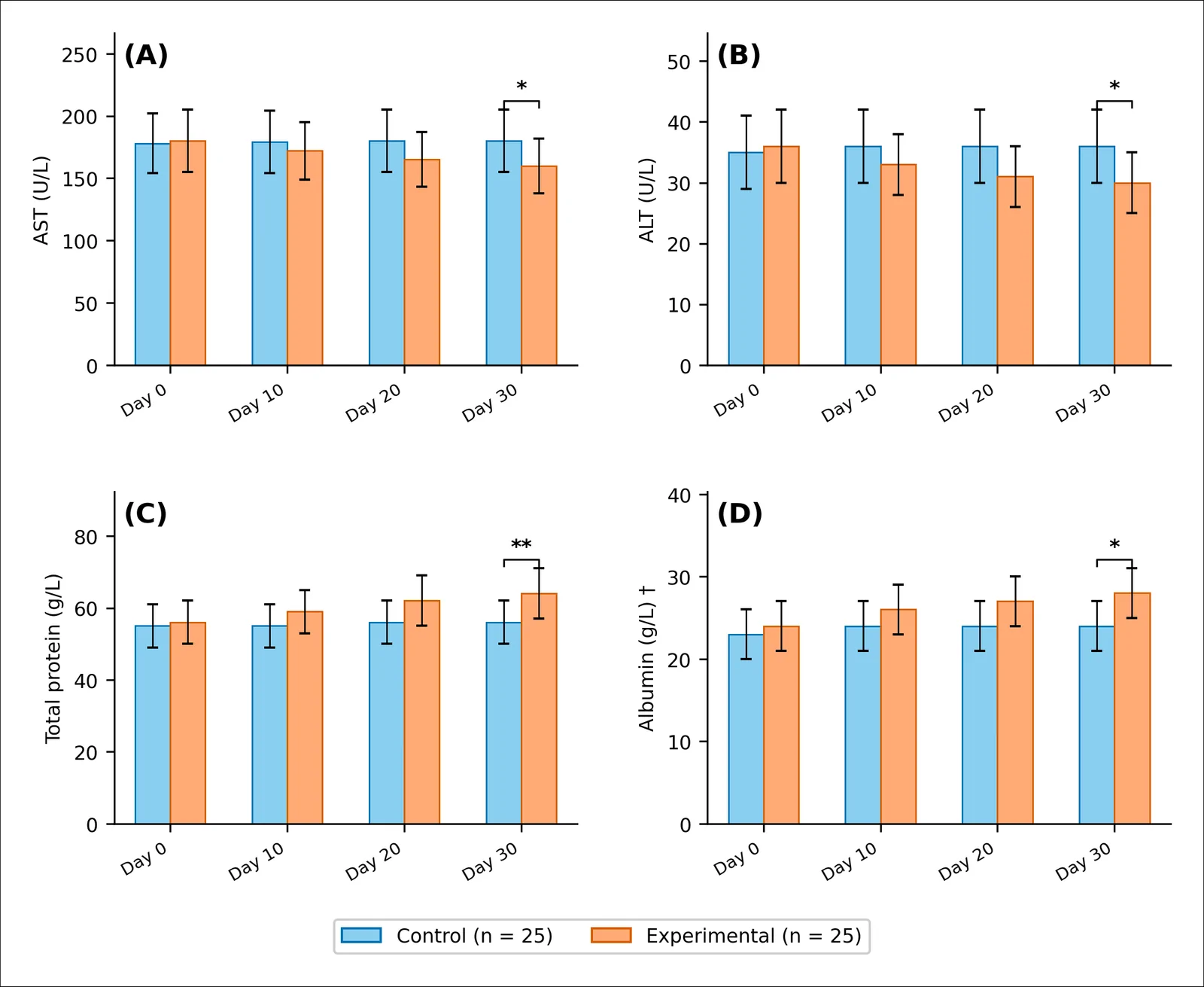
Hepatic biochemical parameters.

### Safety and mortality observations

No birds died, were culled, or were removed from the study during the 30-day experimental period, and all 50 hens completed the trial. No feed refusal or selective feeding was observed, and daily dry matter intake did not differ between groups. No adverse clinical signs were observed during the study, including neurological abnormalities potentially relevant to the thujone-containing *A. absinthium* component.

## DISCUSSION

This trial provides original *in vivo* evidence that a regionally sourced multicomponent mineral–phytogenic supplement can simultaneously influence two stressors that commonly coexist in commercial laying flocks but are usually investigated separately: gastrointestinal helminthiasis and dietary mycotoxicosis. Previous studies have predominantly evaluated individual agents or limited combinations directed toward only one of these stressors [13, 24, 31, 34]. In the present study, a single formulation was associated with concurrent responses across six major endpoint domains, parasitological, toxicokinetic, hematological, hepatic, antioxidant, and productive, measured longitudinally in the same birds under a common experimental framework. This integrated response is a central strength of the study because it allows the effects of supplementation to be interpreted across multiple physiologically relevant systems rather than from a single isolated outcome.

**Figure 5:**
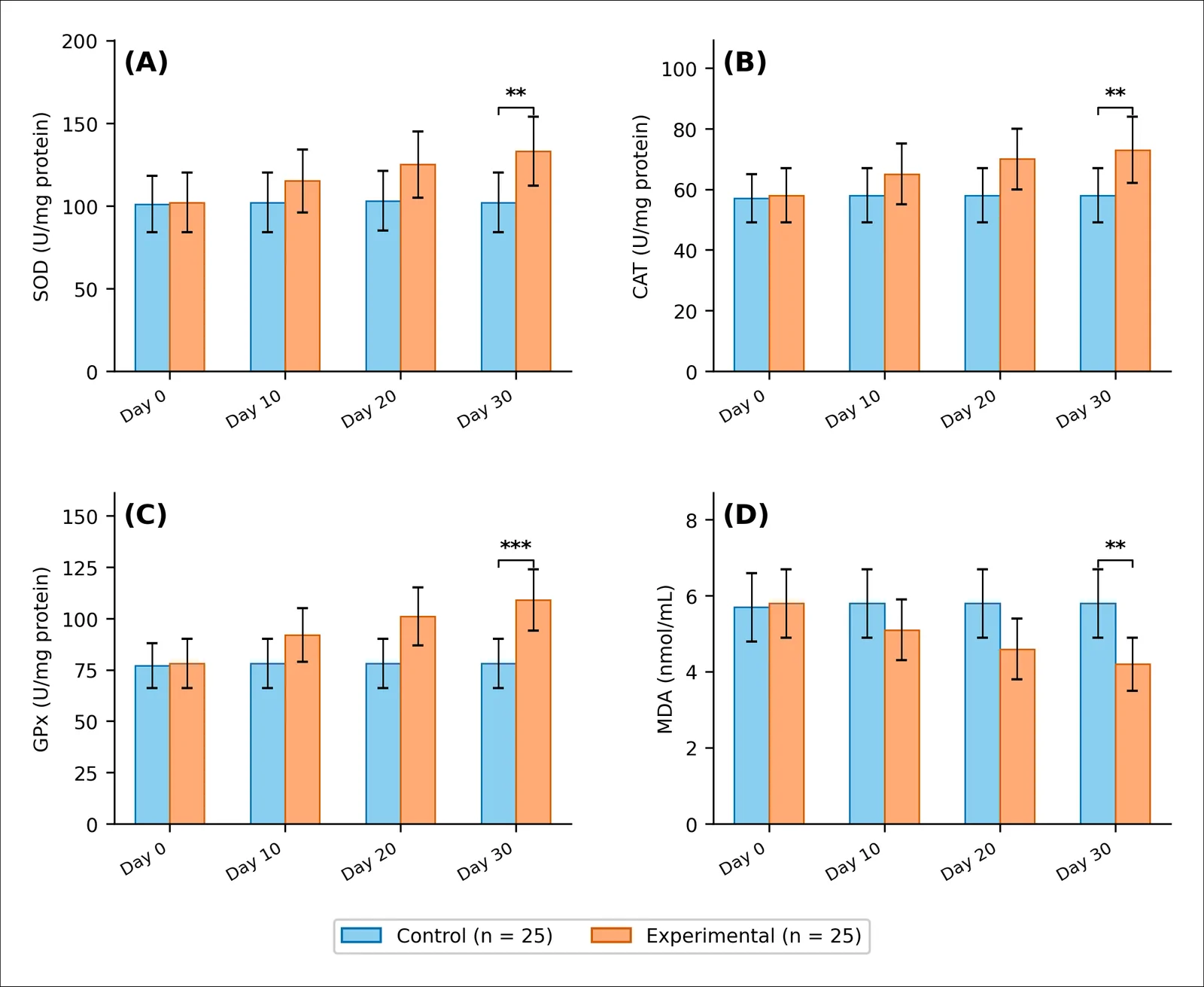
Intracellular antioxidant enzyme activities and lipid peroxidation.

### Parasitological outcomes

Fecal egg shedding declined progressively in the supplemented group, reaching 64.0% below the control level by day 30 (IRR = 0.36; 95% CI = 0.22–0.51). This magnitude of reduction falls within the upper range reported for plant-based preparations evaluated in naturally infected poultry. Ethanolic *Artemisia* extracts have been shown to reduce female worm fecundity, FEC, and worm burdens in infected chicks [35], whereas a seven-plant extract containing *A. absinthium* achieved a 91.3% overall reduction in FEC but did not reach the 90% efficacy threshold specifically for *A. galli* [36]. In laying hens, pumpkin seeds provided at 10 g/bird/day did not produce a measurable parasitological effect [34], and neither pumpkin nor *Artemisia*-based phytogenic preparations reduced worm burdens when evaluated individually at the tested inclusion rates [24].

The stronger response observed with the present multicomponent formulation may therefore reflect complementary actions among the constituent materials rather than the activity of a single ingredient. However, this interpretation is based on comparisons across separate studies and cannot establish synergy or a true matrix effect because the present trial did not include single-component treatment arms [37]. Nevertheless, the sustained reduction in fecal egg shedding may have practical epidemiological relevance because *A. galli* can persist within poultry production systems and contribute to repeated exposure of successive flocks [38].

### Mycotoxin biomarkers

By day 30, circulating AFB1, DON-3S, and T-2 toxin concentrations were 37.9%, 38.8%, and 41.9% lower, respectively, in supplemented birds than in controls. These reductions are broadly comparable with decreases in relative oral bioavailability reported for some experimental decontaminants; for example, an algoclay-based intervention in a broiler oral-bolus model reduced the relative bioavailability of DON, ochratoxin A, and AFB1 by 39.9%, 44.3%, and 64.1%, respectively [39].

An important feature of the present feeding design must be considered when interpreting these reductions. Because the supplement was added to the basal diet at 170 g/kg, the concentrations of all basal dietary constituents, including naturally occurring mycotoxins, were diluted by a factor of 1000/1170 = 0.855. Accordingly, estimated complete-diet concentrations decreased to approximately 17.4 µg/kg AFB1, 4.36 mg/kg DON, and 29.2 µg/kg T-2 toxin. Because dry matter intake did not differ between groups, daily mycotoxin intake would therefore have been approximately 14.5% lower in supplemented birds due to dilution alone. Thus, part of the observed decline in circulating biomarkers can be attributed to reduced dietary exposure, and the remaining reduction may reflect additional effects on adsorption, absorption, metabolism, or a combination of these processes.

The DON-3S response is particularly notable because DON is generally considered difficult to adsorb effectively using conventional mineral binders. A broad evaluation of binders found negligible adsorption of DON [40], whereas bentonites have shown strong binding of AFB1 but only limited adsorption of DON [41]. In the present formulation, opoka represented only 1.5% of the basal diet. Consequently, reduced DON-3S concentrations are unlikely to be explained by mineral adsorption alone. One possible explanation is reduced intestinal absorption associated with improved barrier function, since DON at dietary concentrations relevant to poultry can increase paracellular permeability [42], and higher oral doses have been reported to downregulate ZO-1 and claudin-1 expression in brown laying hens [43]. However, the present study did not measure intestinal permeability or tight-junction expression; therefore, this mechanism remains a biologically plausible interpretation rather than a demonstrated pathway.

### Hematological parameters

Hematological responses were characterized by higher RBC, Hb, and Hct values, together with lower WBC and eosinophil values, in supplemented birds. This pattern contrasts with the hematological disturbances commonly associated with *A. galli* infection, which include reduced erythrocyte indices and increased eosinophilic responses [44]. The eosinophil response was particularly marked, with a 45.2% reduction from baseline compared with a 14.4% reduction in total WBC, and its temporal pattern closely paralleled the decline in fecal egg shedding.

Eosinophils are important cellular components of the host response to gastrointestinal helminths and have been described as prominent early infiltrating leukocytes within the jejunal lamina propria during *A. galli* infection [45]. The concurrent reductions in eosinophils and fecal egg shedding therefore suggest that the parasitological response was accompanied by attenuation of a systemic cellular response associated with helminth infection. However, because tissue-level inflammatory responses and worm burdens were not directly quantified, the hematological changes should not be interpreted as direct evidence of reduced intestinal inflammation.

### Hepatic biochemical responses

Supplemented hens exhibited lower AST and ALT activities, along with higher total protein and albumin concentrations, by day 30. This pattern is opposite to the biochemical profile commonly reported during aflatoxicosis in laying hens, in which AFB1 exposure has been associated with increased hepatic transaminases and MDA and reduced total protein, albumin, and antioxidant enzyme activities [46]. Comparable protective responses have also been reported with multicomponent detoxifying interventions in Lohmann Brown hens exposed to AFB1 and T-2 toxin, including reductions in hepatic and renal toxin deposition [47].

Nevertheless, the observed changes cannot be attributed exclusively to reduced mycotoxin exposure. *A. galli* infection itself has also been associated with decreases in circulating total protein and albumin [44]. Therefore, the higher protein and albumin concentrations in supplemented hens may reflect combined improvement in nutritional, parasitological, and toxicological status. In addition, no histopathological assessment of hepatic tissue was performed, so the reductions in AST and ALT should be interpreted as biochemical evidence of altered hepatic status rather than direct confirmation of reduced hepatic injury.

### Antioxidant status

Supplementation was associated with higher erythrocyte SOD, CAT, and GPx activities and lower serum MDA concentrations. These concurrent changes suggest improved systemic redox balance because antioxidant enzyme activity increased while a marker of lipid peroxidation decreased. Similar responses have been reported with *A. absinthium*-derived interventions; *A. absinthium* essential oil improved erythrocyte antioxidant systems in *Eimeria*-challenged broilers [48], whereas aqueous extracts restored SOD and GPx activity in experimental liver injury models [49].

Increased antioxidant enzyme activity may reflect either enhanced antioxidant capacity or compensatory upregulation in response to persistent oxidative stress. In the present study, however, the simultaneous reduction in MDA supports the interpretation that the antioxidant response was accompanied by lower lipid peroxidation rather than by compensatory enzyme induction alone. Even so, the specific contribution of individual supplement components cannot be determined because the formulation was evaluated as a combined treatment.

### Integrated response of the multicomponent supplement

A major contribution of the present study is the consistency of responses across multiple biological domains. In the same 50 individually housed hens, reduced *A. galli* egg shedding occurred concurrently with lower circulating mycotoxin biomarkers, reduced lipid peroxidation and hepatic enzyme activities, improved erythroid indices, and improved productive performance. Each pathway has been independently linked to one or both experimental stressors. *Ascaridia galli* infection can impair nutrient utilization and alter energy and lipid metabolism [50], DON can compromise intestinal barrier function [42, 43], and AFB1 is associated with hepatic oxidative injury [46]. The simultaneous improvements across these pathways within a single cohort therefore support the concept that a multicomponent nutritional strategy may provide broader physiological benefits than interventions directed at only one stressor.

Researchers have previously investigated combination approaches in poultry exposed to mycotoxins. Protective responses have been reported in ochratoxin-exposed breeders [51], while combinations of binders and phytobiotics have improved performance during aflatoxin exposure [52]. However, the present study did not include single-component treatment arms or an isocaloric placebo control. Consequently, the trial compares two complete dietary regimens rather than isolating the independent contribution of the supplement itself or each constituent component. Consider this limitation when interpreting the magnitude and mechanism of the observed effects.

### Egg production and egg quality

The improvements in egg production and egg quality likely arose through more than one mechanism. Yolk color showed the largest relative change among the production-related endpoints, increasing markedly in supplemented hens. This response is plausibly related to the carotenoid-rich pumpkin pulp and peel fraction of the diet. A meta-analysis of 47 studies reported a pooled improvement of 2.11 yolk color units (95% CI = 1.71–2.51) following dietary carotenoid supplementation [53]. Therefore, the increase in yolk pigmentation observed here is consistent with the known pigmentary effects of carotenoid-containing feed ingredients.

The improvements in shell thickness and shell breaking strength are less readily explained by a direct pigmentary or simple mineral-supply effect. The same meta-analysis found no consistent pooled effect of carotenoid supplementation on eggshell thickness [53]. In the present study, estimated calcium supply was also similar between diets because calcium contributed by coquina meal was largely offset by dilution of the basal diet, resulting in approximately 35.5 versus 35.2 g Ca/kg. Improved calcium utilization could therefore be considered as one possible explanation. Interventions that improve villus architecture and intestinal absorptive function in laying hens have been associated with increased calcium retention, calbindin-D28k expression, and improved eggshell characteristics [54, 55]. Relief of mycotoxin-related effects on intestinal or reproductive function may also have contributed [47, 51]. However, intestinal morphology, calcium absorption, calbindin expression, and shell-gland function were not measured; therefore, these mechanisms remain speculative.

Supplemented hens also consumed approximately 10 kcal ME/day less than controls while producing approximately 7.1 g/day more egg mass, corresponding to approximately 14 kcal/day of additional egg output. This pattern is consistent with improved productive efficiency and may reflect reduced physiological costs associated with parasitic infection and mycotoxin exposure [50, 56]. However, because feed energy density differed between the two complete diets and no isocaloric control was included, interpret this observation cautiously and do not consider it direct evidence of improved energetic efficiency attributable solely to the supplement.

## CONCLUSION

The present study demonstrated that 30-day supplementation with a regionally sourced multicomponent mineral–phytogenic formulation was associated with concurrent improvements in parasitological, toxicological, hematological, hepatic, antioxidant, and productive outcomes in naturally challenged laying hens. By day 30, *A. galli* fecal egg shedding was 64.0% lower than in controls (IRR = 0.36; 95% CI = 0.22–0.51), while circulating AFB1, DON-3S, and T-2 toxin concentrations were reduced by 37.9%, 38.8%, and 41.9%, respectively. Supplemented hens also showed improved RBC, Hb, and Hct values; lower WBC and eosinophil values; lower AST, ALT, and MDA; and higher total protein, albumin, SOD, CAT, and GPx. These physiological responses were accompanied by increased hen-day production, egg weight, daily egg mass, shell thickness, shell breaking strength, and yolk color. No mortality, feed refusal, selective feeding, or adverse clinical signs were observed during the trial.

From a practical perspective, the findings indicate that a formulation based on opoka, coquina meal, *C. maxima*, and *A. absinthium* may offer a nutritional approach to simultaneously address two challenges that frequently coexist in poultry production: gastrointestinal helminth infection and dietary mycotoxin exposure. Using regionally available and physicochemically characterized raw materials may also support formulation reproducibility and could be particularly relevant where dependence on imported feed additives is economically restrictive. A major strength of the study was the longitudinal evaluation of multiple endpoint domains in the same individually housed birds under natural *A. galli* infection and naturally occurring multi-mycotoxin exposure. Random allocation, blinded laboratory assessments, repeated sampling, and the inclusion of both health-related and productive outcomes further strengthened the experimental framework.

Several limitations should nevertheless be considered. The study included 50 hens from a single age-matched flock and was limited to a 30-day experimental period and one supplementation level. The absence of single-component treatment groups prevents determination of the independent contribution or potential interaction of the mineral and phytogenic constituents. In addition, the experimental and control diets were not isocaloric, and supplementation diluted basal diet mycotoxin concentrations by approximately 14.5%; therefore, the reductions in circulating biomarkers cannot be attributed entirely to sorption or altered absorption. The study did not evaluate direct adult worm burdens, intestinal histomorphology, intestinal permeability, tight-junction expression, hepatic histopathology, calcium absorption, or shell-gland responses, limiting mechanistic interpretation.

Future studies should therefore include larger and independent laying-hen populations, multiple farms or flocks, longer observation periods, and dose–response designs. Factorial studies incorporating individual supplement components and an appropriately matched isocaloric control would help distinguish additive, complementary, or synergistic effects. Direct quantification of intestinal worm burdens, intestinal morphology and barrier function, hepatic pathology, mycotoxin toxicokinetics, mineral adsorption capacity under gastrointestinal conditions, calcium utilization, and persistence of production responses would further clarify the mechanisms underlying the observed effects. Evaluation under commercial production conditions would also be required to determine reproducibility, economic feasibility, long-term safety, and practical applicability.

Overall, the coordinated reduction in *A. galli* shedding and systemic mycotoxin biomarkers, together with improvements in physiological status and egg production, supports further development of this multicomponent mineral–phytogenic formulation as an integrated nutritional strategy for laying hens exposed to concurrent parasitic and mycotoxin challenges. However, larger, longer-term, mechanistically controlled trials are warranted before the formulation can be considered a replacement for established anthelmintic or mycotoxin-management programs.

## DATA AVAILABILITY

The data generated during the study are included in the manuscript.

## GENERATIVE AI DECLARATION

The authors declare that generative artificial intelligence (AI) tools were used solely to improve language, grammar, and readability during manuscript preparation. All scientific content and conclusions were developed and verified by the authors. The authors take full responsibility for the accuracy, integrity, and originality of the work presented, and no AI tool was listed as an author.

## AUTHORS’ CONTRIBUTIONS

NM: Conceptualization, investigation, and manuscript drafting. SM: Methodology and investigation. NM and AS: Data collection and laboratory analyses. AK: Supervision, statistical analysis, manuscript review, and editing. All authors read and approved the final manuscript.
